# The Profiling of DNA Methylation and Its Regulation on Divergent Tenderness in Angus Beef Cattle

**DOI:** 10.3389/fgene.2020.00939

**Published:** 2020-08-26

**Authors:** Chunping Zhao, Guanyu Ji, José A. Carrillo, Yaokun Li, Fei Tian, Ransom L. Baldwin, Linsen Zan, Jiuzhou Song

**Affiliations:** ^1^College of Animal Science and Technology, Northwest A&F University, Xianyang, China; ^2^Department of Animal and Avian Sciences, University of Maryland, College Park, MD, United States; ^3^Shenzhen GenDo Health Sci&Tech Ltd., Shenzhen, China; ^4^Animal Genomics and Improvement Laboratory, BARC, NEA, USDA, Beltsville, MD, United States

**Keywords:** DNA methylation, MBD-seq, DMR, epigenetic, beef quality

## Abstract

Beef is an essential food source in the world. Beef quality, especially tenderness, has a significant impact on consumer satisfaction and industry profit. Many types of research to date have focused on the exploration of physiological and developmental mechanisms of beef tenderness. Still, the role and impact of DNA methylation status on beef tenderness have yet to be elucidated. In this study, we exhaustively analyzed the DNA methylation status in divergent tenderness observed in Angus beef. We characterized the methylation profiles related to beef tenderness and explored methylation distributions on the whole genome. As a result, differentially methylated regions (DMRs) associated with tenderness and toughness of beef were identified. Importantly, we annotated these DMRs on the bovine genome and explored bio-pathways of underlying genes and methylation biomarkers in beef quality. Specifically, we observed that the ATP binding cassette subfamily and myosin-related genes were highly methylated gene sets, and generation of neurons, regulation of GTPase activity, ion transport and anion transport, *etc.*, were the significant pathways related with beef tenderness. Moreover, we explored the relationship between DNA methylation and gene expression in DMRs. Some methylated genes were identified as candidate biomarkers for beef tenderness. These results provide not only novel epigenetic information associated with beef quality but offer more significant insights into meat science, which will further help us explore the mechanism of muscle biology.

## Introduction

Beef constitutes one of the primary food sources worldwide due to its high-quality protein and other nutrients, such as B vitamins, iron, zinc, unsaturated fatty acid, *etc.* (Delgado, 2003; McNeill and Van Elswyk, 2012). Thus, beef composition and quality have always been a principal focal point for both consumers and producers (Galbraith, 2002; Raes et al., 2003). Traditionally, beef quality has been influenced and evaluated by three general criteria: tenderness, juiciness, and flavor (Boleman et al., 1997), of which, juiciness and flavor are subjective and influenced by several factors, such as cooking style, consumer preferences, and ethnic, cultural habits. However, tenderness is one of the most important factors influencing the quality and can be quantitatively and objectively measured by the Warner–Bratzler shear forces (WBSF) (Huffman et al., 1996; Ferraris et al., 2013; Hocquette et al., 2013; Morales et al., 2013; Nogalski et al., 2018). Tenderness is a complex trait influenced by different structural components, such as the myofibrillar mass, sarcoplasmic proteins, intramuscular fat, and connective tissue, *etc*. Besides, many intricate biological processes are also involved, such as rigor development, fiber contraction, proteolysis during aging and meat maturation (Tornberg, 1996; Patten et al., 2008; Nishimura, 2010).

To date, a large number of molecular studies have focused on the exploration of molecular mechanisms responsible for beef tenderness (Zhao et al., 2012a,b, 2014, 2015; Picard et al., 2015; Silva et al., 2016; Enriquez-Valencia et al., 2017; Goncalves et al., 2018; Gagaoua et al., 2019; Leal-Gutierrez et al., 2019). Interestingly, these studies revealed some factors related with beef tenderness and meat quality, such as quantitative trait loci (QTL), single nucleotide polymorphisms (SNPs), copy number variations (CNVs), functional candidate genes, transcripts, and proteins, *etc.* (Zhao et al., 2012b, 2014; Silva et al., 2016; Enriquez-Valencia et al., 2017; Goncalves et al., 2018; Gagaoua et al., 2019; Leal-Gutierrez et al., 2019). In our previous researches, we ascertained that some genes, proteins, miRNAs, and histone modification, namely an epigenetic factor, are demonstrably related with beef tenderness and meat quality (Zhao et al., 2012a,b, 2014, 2015). However, DNA methylation has not been elucidated in the regulation of beef quality traits.

DNA methylation is one of the most studied epigenetic modifications which occurs by covalently attaching a methyl group to the fifth carbon of cytosine residues (Goldberg et al., 2007). It is reported that DNA methylation plays essential roles in diverse processes, including cell proliferation and differentiation, genomic imprinting, embryogenesis, X chromosome inactivation, tumor genesis, *etc.* (Wilson, 2008). Moreover, DNA methylation has been associated with gene repression at specific genome regions and mainly when it occurs in gene promoters (Mendoza-Garces et al., 2013). Much attention has been paid to the figuration of DNA methylation on the whole genome in livestock animals, such as chicken, pig, horse, beef cattle and dairy cattle using methylated DNA immune-precipitation sequencing (MeDIP-seq) (Li et al., 2011, 2012; Huang et al., 2014; Lee et al., 2014; Song et al., 2016); goat and beef cattle using methylated DNA binding domain-sequencing (MBD-seq) (Frattini et al., 2017; Li et al., 2019); sheep using reduced representation bisulfite sequencing (RRBS) (Couldrey et al., 2014), hen using whole-genome bisulfite sequencing (WGBS) (Zhang et al., 2017), etc. These studies identified differentially methylated regions (DMRs) and methylation-regulated genes related to variant phenotypes or different development stages in domestic animals. Intriguingly, one of the studies found that DNA methylation was related to meat quality and tenderness of breast muscle in chicken (Li et al., 2011). However, the DNA methylation analysis of beef quality and tenderness remains unknown.

In the present study, we explored DNA methylation in *longissimus lumborum* with divergent tenderness of inbred Angus cattle. We first depicted the methylation profiles related to beef tenderness and described methylation distributions on the whole genome. We then identified DMRs between those divergent tenderness beef and annotated the DMRs on the bovine genome and subsequently explored the bio-pathways with those underlying genes of DMRs. Finally, we examined the relationships between gene expression and these selected methylated markers. We believe that the results of the study will contribute to excavating epigenetic mechanisms regulating beef quality and provide valuable information for further functional validation and, ultimately, promote the improvement of beef production.

## Materials and Methods

### Sample Preparation and Experimental Design

Nineteen Angus cattle were obtained from WYE Angus (Queenstown, MD, United States). They consisted of contemporaneous steers that received the same pelleted forge diet formulated to provide the nutritional requirement. Around 12 months of age, the animals were terminated, and samples of *longissimus lumborum* from the 12–13th rib were collected and aged at 4°C for 14 days. Concurrently, a small piece of fresh tissue samples from the same muscle was obtained and immediately placed in RNAlater solution at −80°C for DNA and RNA extraction. After the aging process, WBSF, crude fat, fatty acid contents, and cooking loss of the samples were measured to evaluate beef tenderness as previously described (Zhao et al., 2012b). Then four samples exhibiting the lowest WBSF were selected as the tender group and four individuals with the greatest WBSF designed as the tough group.

All procedures were approved by the University of Maryland Institutional Animal Care and Use Committee (Protocol # R-07-05). Research performed in this study were in accordance with the relevant guidelines and regulations of the ethics approvals above.

### DNA Extraction and MBD-Seq Library Preparation

Two samples were randomly selected as representatives from four individuals of each group (tender and tough), and Genomic DNA was extracted using the Wizard Genomic DNA purification kit (Promega, Madison, WI, United States). DNA concentrations were measured by the Qubit dsDNA Broad-Range Assay (Invitrogen, Carlsbad, CA, United States). The MBD-seq method was used to identify methylated DNA regions, and elution of the captured methylated DNA was done separately using three salt gradients, namely the low, medium and high concentrations as our previous publication (Carrillo et al., 2015). MethylCap kit (Diagenode, Denville, NJ, United States) was used to obtain DNA containing methylated CpGs. Briefly, DNA was extracted and sheared into 300–500 bp fragments using the Bioruptor^®^ Sonicator (Diagenode) and then visualized on an agarose gel to verify the size of the resultant segments. A 141.8 μl capture reaction mix containing 12 μl of sheared DNA and lacking the MethylCap protein was prepared. From this preparation, 119 μl of capture reaction mix was incubated with 1 μl of diluted MethylCap protein at 40 rpm on a rotating wheel for 2 h and at 4°C to allow the interaction. The remaining solution (22.8 μl) was used as an input sample. Magnetic beads captured methylated DNA. Unbound DNA was washed off, and the eluted DNA collected. For the elution, 150 μl of the low, medium, and high concentration elution buffer was used serially per capture. These three elutions are corresponding to the high, medium, and low methylation parts of the whole genome, respectively (Brinkman et al., 2010). They were named as HTE (High methylated-DNA from tender beef), HTO (High methylated-DNA from tough beef), MTE (Medially methylated-DNA from tender beef), MTO (Medially methylated-DNA from tough beef), LTE (Low methylated-DNA from tender beef), LTO (Low methylated-DNA from tough beef), respectively. All fractions and input were purified using the MiniElute PCR Purification Kit (QIAGEN, Valencia, CA, United States). Quantitative PCR (qPCR) (iCycleriQ PCR system, Bio-Rad, Hercules, CA, United States) was performed in duplicate for each sample to test the enrichment efficiency. Method 2^–ΔΔCt^ was applied to determine relative fold enrichments and compared enrichment values of a positive *TGFB3* to a negative primer pair *MON2*, between experimental (methyl DNA) and reference (input DNA) samples.

The sequencing libraries were constructed as follows: NEBNext^®^ End Repair Module (NEB, Ipswich, MA, United States) was used for the end repair of the fragmented methylated DNA, a 3′ A was added using DNA Polymerase I, Large (Klenow) Fragment (NEB), then a pair of Solexa adaptors (Illumina, San Diego, CA, United States) was ligated to the repaired ends by T4 ligase (Promega). Filtration in a 2% agarose gel was used to select fragments (DNA plus adaptors) from 300 to 500 bp. PCR enriched purified DNA templates were amplified by Phusion^®^ Hot Start High-Fidelity DNA Polymerase (NEB). After purification, DNA quality was examined. The DNA library was diluted, and the concentration double-checked using the Qubit assay (Life Technologies). A total of 12 libraries were constructed for three gradient-eluted DNA from two replicates of two groups. Finally, the libraries identified by the 6-bp index were sequenced at 50 bp sequence read using an Illumina HiSeq 2000 sequencer.

### MBD-Seq Data Analysis

The raw reads were obtained by Illumina sequencing. We created the clean reads from the dataset by filtering reads contained the adaptor sequence or the reads which had low quality and N bases occupy more than 50% of the read length (Chen et al., 2018). The clean reads were obtained by SOAPnuke and aligned to the cattle reference genome (BosTau8) using the Burrows-Wheeler Aligner (BWA) (Li and Durbin, 2010), allowing up to two nucleotide mismatches to the reference genome per seed and returning only uniquely mapped reads. Replicate sequencing reads (i.e., reads with the same starting position) were counted only once.

Uniquely mapped reads were used to analyze the DNA methylation differential peaks based on a defined model of ChIP-Seq data (MACS2) (Liu, 2014). Peaks were identified by the Poisson test with *p*-value < 0.01; then, we modified results by FDR < 0.01 for further analysis, and all the other parameters were used as the default (Wang et al., 2018).

We found most of the peaks in the biological replicates overlapped with the merged peaks. Thus, we determined that the data may not be saturated and selected data for the next differential analysis. The peaks from the tender and tough samples were then merged as candidate differentially methylated regions (CDMRs). The reads were counted for each of the CDMRs, and the DESeq Bioconductor package (Anders and Huber, 2010) was used to identify CDMRs with an FDR < 0.01 and |log_2_ FC| > 2 as final differentially methylated regions (DMRs). Read distributions were normalized using the Reads Per Kilobase per Million (RPKM) mapped reads strategy in 100 bp bins, followed by the analysis of the immune-precipitation-based DNA methylome using a method based on a Bayesian deconvolution strategy (Down et al., 2008). A prior report (Chavez et al., 2010) demonstrated that this normalization strategy improves the correlation to bisulfite sequencing data.

The paired reads were not extended and were placed in bins of 100 bp across the genome. The values of each bin were used to determine the read density or read distribution on the gene element regions. All the details on data analysis can be found in a previous publication (Wang et al., 2018).

### Joint Analysis of MBD-Seq and Gene Expression

Gene expression of the four samples in each group (tender and tough) was assessed with the 4 × 44K Bovine Gene Expression Microarray (Agilent, Santa Clara, CA, United States) as previously described (Zhao et al., 2012b). Pearson correlation was used for correlation analysis between DNA methylation and gene expression. Specifically, the reads density [uniformed by tags per million (TPM) method] of the peaks, which located in the region from transcription start site (TSS) upstream 2000 bp to downstream 2000 bp, were statics to represent the degree of DNA methylation on the genes. Then, three levels of DNA methylation for tender and tough samples were totally counted separately. At last, the difference values between the tender and tough for the three levels of DNA methylation were pairing with the difference of gene expression (beta value for microarray were also uniformed by TPM method) respectively, and cor.test() in the default functions of R package was used to calculate the Pearson correlation coefficient with default parameters. Specifically, the parameter alternative with two.sided, conf.level = 0.95. The difference value of the DNA methylation and the difference of gene expression were performed by scatter plot.

## Results

### The Meat Quality of *Longissimus Lumborum* From Angus Cattle

A total of 19 Angus cattle were slaughtered, and *longissimus lumborum* samples were collected. The measurement of meat quality traits showed that WBSF values differed significantly for these 19 steers, ranged from 5.81 to 20.70 kg (13.37 ± 5.35 kg), but crude fat, fatty acid contents, and cooking loss varied slightly. To explore the mechanisms of tenderness variation, four samples exhibiting the lowest WBSF (6.03 ± 0.31 kg) were selected to represent the tender group and four samples with the greatest WBSF (20.33 ± 0.53 kg) were designated as the tough group. After further analysis of the meat quality traits between tender and tough groups, we found that crude fat content was also significant between these two groups (*P* < 0.05).

### The DNA Methylation in *Longissimus Lumborum* Muscle of Angus Cattle

To detect the distribution of DNA methylation across the entire genome of *longissimus lumborum* from Angus, MBD-seq was performed in the beef with divergent tenderness, namely tender and tough. By using deep sequencing, we obtained a total of 146271818 raw reads. After filtering the reads of low quality, we aligned those clean reads to the reference genome (bosTau8), and our results reveal that 92.25% of reads were uniquely mapped to the reference (Supplementary Table S1). The uniquely mapped reads were 35257025, 52946431, and 46738093 in the high, medium, and low methylated regions, respectively, implying that more proportion of the genome in *longissimus lumborum* of Angus is moderately methylated. Additionally, a total of 63346070 uniquely mapped reads were identified in the tender group while 71595479 in the tough samples (Supplementary Table S2).

**FIGURE 1:**
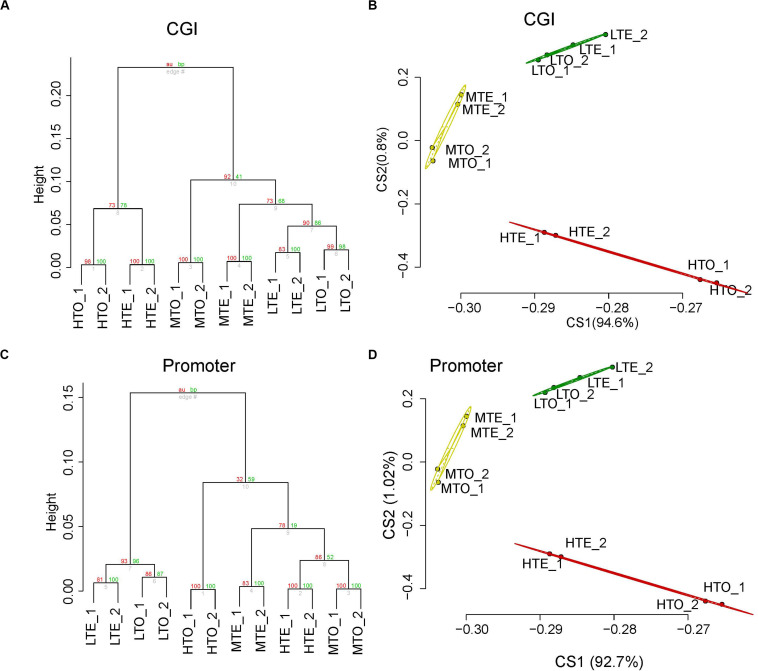
Methylation profile analysis of *longissimus lumborum* from cattle on CGI and promoter. **(A)** Unsupervised hierarchical clustering analysis on CGI. **(B)** Principal Component Analysis on CGI. **(C)** Unsupervised hierarchical clustering analysis on promoter. **(D)** Principal Component Analysis on promoter.

To check the distribution of DNA methylation, CpG islands (CGIs), promoters, namely the most methylated and functional regions in the genome, were partitioned; average distance cluster analysis was carried out using uniquely mapped reads in these areas normalized with RPKM. All regions were divided into several clusters so as to present differential DNA methylation patterns in CGIs and promoters subtly. The cluster analysis results are shown in Figures 1A,C, in which, every two replicates are tightly clustered in the bottom of the dendrogram, and some regions have been overtly divided according to methylation levels. All the high methylated regions were distinctly separated from others, and all the low methylated regions also cluster together in CGIs (Figure 1A). In contrast, the DNA methylation of middle and high has closer distance in promoter regions (Figure 1C).

To further verify the results of cluster analysis, Principal Components Analysis (PCA) was also performed in CGIs and promoters, respectively. As shown in Figures 1B,D, after the analysis of dimension reduced, the top two principal components could distinguish those two elements clearly. The three clusters, namely the high, medium, and low methylated regions, separated along with the first two principal components. Additionally, the two biological replicates were highly consistent in read density in CGIs and promoters, and they had a closer distance in the cluster and PCA. Therefore, the reads from the two replicates in the same condition were merged respectively for further analysis.

### DNA Methylation Profiles of *Longissimus Lumborum*

**FIGURE 2:**
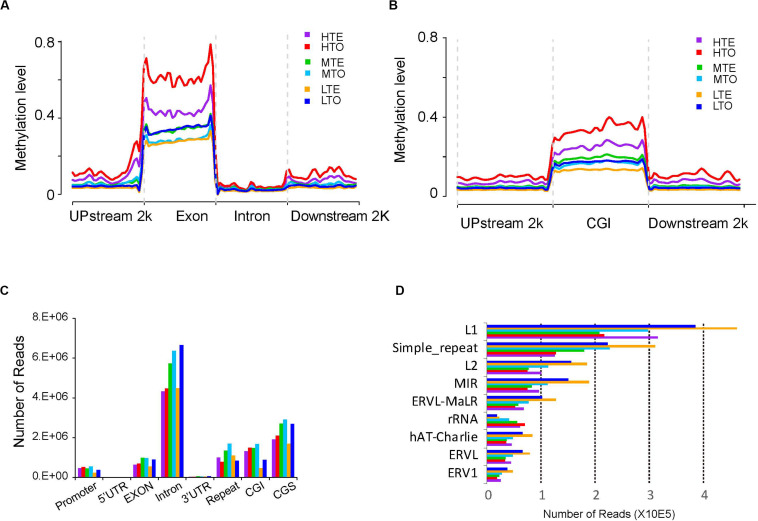
Methylation levels and read distributions in each gene elements and repeat elements. Reads were normalized by RPKM (Reads Per Kilobase per Million mapped reads). **(A)** Distribution of 5-mC tag densities on genome. **(B)** Distribution of 5-mC densities on CGIs. **(C)** Read distribution on genome elements. **(D)** Read distribution in repeat elements.

To obtain the global DNA methylation landscapes in the *longissimus lumborum*, the methylation levels were explored in different genome regions, including the upstream 2 kb, exon, intron, and downstream 2 kb of genes, after the read number was normalized using RPKM method. As shown in Figure 2A, the methylation levels dramatically increased in the exons. On the whole genome, the exons showed a much higher methylation level than the other regions, and the global methylation levels of upstream, downstream, intron decreased in turn. Additionally, the methylation levels of HTE and HTO were much higher than those from the medium or low methylated regions.

As reported previously, DNA methylation often occurs in the CGIs of the genome. Therefore, we divided the genome into three parts, namely CGIs, upstream 2 kb of CGIs, and downstream 2 kb of CGIs, to clarify the relationship of DNA methylation and the CpG density. As shown in Figure 2B, the methylation levels were found to increase sharply in the CGIs compared to upstream and downstream. Three levels of methylation were successively high (HTE and HTO), medium (MTE and MTO), and low (LTE and LTO), from top to bottom.

To explore the methylation levels on the different components of genes, we examined the read distribution in the promoter, 5′UTR, exon, intron, 3′UTR, repeat elements, CGIs, and CpG shores (CGSs). The introns were observed to have enriched with more unique reads than the other components, and CGSs harbored with more reads than CGIs (Figure 2C). Moreover, the 5′ UTR and 3′ UTR were seldom methylated compared to the other components, while numerous reads enriched in the repeat elements. Further, the tough always had more reads than the tender in every element, except in repeat elements, where HTE had more reads than HTO, and LTE had more reads than LTO. Thus, we further explored the distribution of reading in repeat elements. The results showed that LINE/L1, simple repeat, LINE/L2, SINE/MIR exhibited more enriched unique reads (Figure 2D). And the read distribution pattern in rRNA was different from other repeat elements, namely that in rRNA most-read enriched in HTO, but in other repeat elements, most reads fell in LTE.

**FIGURE 3:**
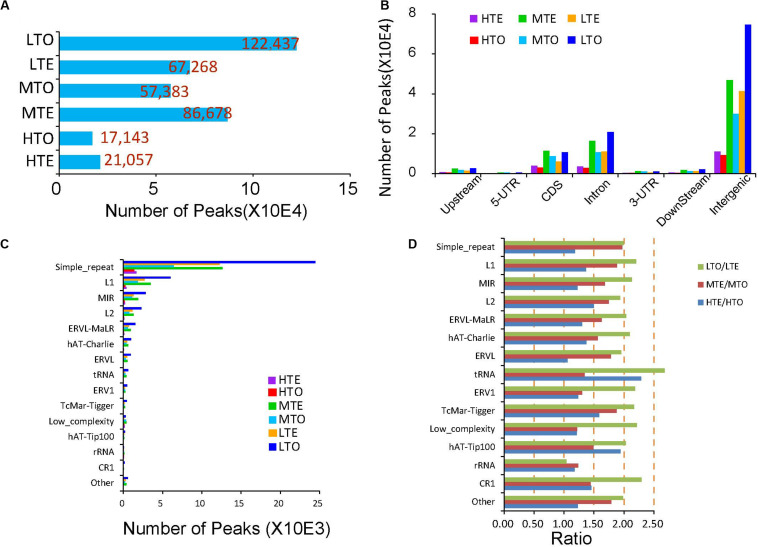
Peak distribution in the tender and tough beef. **(A)** Peak numbers in each elute. **(B)** Peak number distribution on gene elements. **(C)** Peak number distribution in repeat elements. **(D)** Comparison of peak numbers in repeat elements between tough and tender beef.

Then DNA methylation enriched regions, namely peaks, were identified through MACS2, a common approach for clustering to enable identification of enriched domains from ChIP-seq data. The details of the peak numbers are shown in Figure 3A. More peaks were identified in the low methylated regions (LTE and LTO), and the tough (LTO) enriched more peaks than the tender (LTE) in the low methylated areas, which is the inverse of that observed in the medium and high methylated regions. Additionally, in the tender group, the medium methylated regions (MTE) harbored more peaks (86,678), while in the tough group, the low methylated regions (LTO) enriched more peaks (122,437), implying distinct methylation patterns in these two kinds of beef.

We further explored the distribution of peaks in the different components of the genes in the order of upstream 2 kb, 5′UTR, CDS, intron, 3′UTR, downstream 2 kb, and intergenic region. If a peak spans two components, it is only annotated in the prior one. The peak distribution analysis results showed that around 60% of the peaks are located in the intergenic regions. About 15% of peaks are distributed in the introns and CDS, respectively. The peaks in the upstream, 5′UTR, 3′UTR, and downstream are very scarce (Figure 3B). Besides, we refined the peaks distributed in the repeat elements and observed that more than 55% of peaks in the repeat elements are located in simple repeat and 14.01% and 7.23% of peaks in LINE/L1 and SINE/MIR, respectively. Peaks are seldom identified in the other repeat elements (Figure 3C). Then to precisely compare the peaks between the tender and tough we calculated the peak ratios in the repeat elements between the two kinds of beef. As shown in Figure 3D, in the low methylated zones, more than twofold peaks were observed in the tough in all repeat elements except rRNA with an opposite trend for the medium methylated zones. In the high methylation regions, peak numbers were more equalized between tender and tough groups except for tRNA and DNA/hAT-Tip100, *etc*.

### DNA Methylation Variations Between the Tender and Tough Beef

**FIGURE 4:**
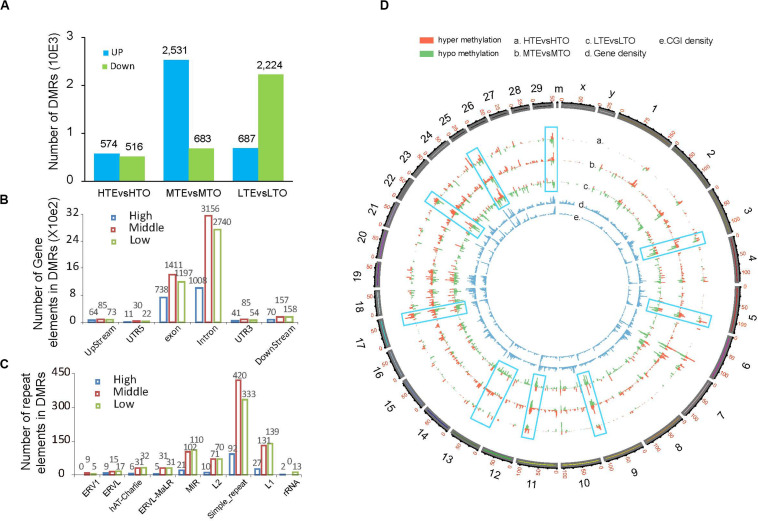
DMR distribution between the tender and tough beef. **(A)** Number of DMRs in each elute. **(B)** Number of DMRs in gene elements. **(C)** Number of DMRs in repeat elements. **(D)** Global DMR distribution on the whole chromosome.

To explore the relationship between DNA methylation and beef tenderness, we identified differentially methylated regions (DMRs) between the tender and tough groups. A total of 7215 DMRs were identified in three methylated levels. In the high methylated regions, almost half of the 1090 DMRs were up-methylated in the tender beef compared with the tough beef; in the MTE 2531 DMRs were up-methylated and 683 DMRs were down-methylated; in the LTO much more down-methylated DMRs were identified in the tough beef (Figure 4A). By separating the peak distribution on the gene components and the repeat elements, we observed more peaks harbored in the MTE for all gene components (upstream, 5′ UTR, exon, intron, 3′ UTR, downstream), while more peaks are enriched in the LTO in all repeat elements except simple repeat (Figures 4B,C).

To graphically demonstrate global DNA methylation variations between these two kinds of beef, a macroscopical display of DMRs along chromosomes was represented by a Circos histogram (Figure 4D), the inner two cycles represent CGI density and gene density respectively, and the outer cycle represents all chromosomes of a cow with scale at 1 Mb bins. We found that the high CGI density regions also predominately harbored more DMRs in the whole genome. Additionally, the methylation patterns on chromosomes were different among these three methylation levels. For example, in the low methylation level (circle c), DMRs on chromosome 13 were hypomethylated while they were hypermethylated in the medium level (circle b). In contrast, DNA methylation variation patterns between the low and high methylation levels on chromosome 17 were the opposite. Furthermore, in the low methylation level of chromosome 3, 9, 22, 29, DMRs were hypomethylated while methylation patterns were similar in the high and medium methylation levels. Thus, variations in DNA methylation exhibited by the different methylation levels may play distinct roles in beef tenderness.

### The Function Annotation of DMRs Between the Tender and Tough Beef

To explore more deeply the putative mechanisms and biological functions of these DMRs, we identified the DMRs underlying genes. Among these annotated genes, we identified more enrichments in ATP binding cassette subfamily and myosin-related genes, such as *ABCA1*, *ABCG1*, *ABCA7*, myosins, myosin heavy chains, myosin light chains, *etc*, which were reported to be involved in fatty acid metabolism and beef tenderness. We annotated these genes by GO term and KEGG pathway analysis. A total of 1242 genes are mapped to the unique Entrez Gene IDs in the high methylated regions, and 960 genes are mapped in the medium and 2154 in the LTO, respectively. The GO term results are interpreted to indicate that DNA methylation differences affect the different biological processes, cellular components, and molecular functions (Supplementary Figure S1). In the biological process, DMRs in the high methylated zones involved the generation of neurons, regulation of GTPase activity, *etc.*, and in the medium methylated zones, DMRs functioned in ion transport and anion transport, *etc.*, while in the low methylated zones DMRs played roles in ion transmembrane transport and generation of neurons, *etc*. The KEGG analysis results indicated that the most highly affected pathways for the high and low methylation regions were focal adhesion, Axon guidance, while regulation of actin cytoskeleton was the most important pathway in the medium methylation regions.

### The Relationship of DNA Methylation and Gene Expression in the Tender and Tough Beef

To examine the relationship between DNA methylation and gene activity in the variation of beef tenderness, we evaluated the relative gene expression rate of tender and tough groups using Agilent 4 × 44K bovine microarray. Subsequently, we combined gene expression data with the MBD-seq results. To visualize the relationship on the whole genome, we divided the genome into upstream, exon, intron, and downstream. And we grouped all genes into four categories according to their relative expression (high, medium, low, and silent) and plotted the distribution of DNA methylation in four parts of the genome for each expression category. The results confirmed that the density of methylation in the exons was higher than the introns, downstreams, and upstreams. Additionally, for these four expression categories, genes with lower expression exhibited a higher density of methylation in exons, indicating that the methylation level of exons negatively correlates with gene expression (Supplementary Figure S2).

**FIGURE 5:**
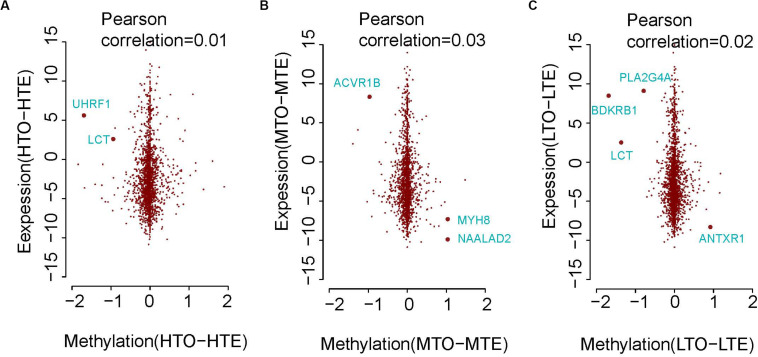
The relationship of methylation and gene expression between the tender and tough beef. **(A)** In high methylation regions. **(B)** In medium methylation regions. **(C)** In low methylation regions.

Previous research found that methylation on the promoter of genes repressed gene expression. Thus, we plotted the scatter diagram to visualize the relationship of DNA methylation in the promoters and gene expression of these two kinds of beef. Although correlations are very low between gene expression and DNA methylation of the promoters as shown in Figure 5, some genes, such as *MYH8, UHRF1*, *LCT, ACVR1B*, *NAALAD2, PLA2G4A*, *BDKRB1*, and *ANTXR1* were identified to exhibit an inverse relationship between expression and methylation. Therefore, these genes may be used as biomarkers of DNA methylation regulating beef tenderness.

## Discussion

Tenderness is one of the most critical factors influencing beef palatability and consumers willingly pay a higher price for beef with guaranteed tenderness. Therefore, consumers’ sensory to beef tenderness is a crucial factor in making a purchase decision (Van Wezemael et al., 2014). Tenderness can be evaluated by sensory methods or instrumental methods. Neural methods assessed by expert panels or untrained consumers are time-consuming and expensive. Therefore, there have been many efforts to devise instrumental methods for determining beef tenderness. The most widely used and accepted method is the Warner–Bratzler method because of programming operation and objective results (Yancey et al., 2010). There is a moderate relationship between Warner–Bratzler shear and sensory assessment of beef tenderness (Destefanis et al., 2008; Wright et al., 2018).

Meat quality is a complex trait controlled by many factors, including genetics, epigenetics, and environments. DNA methylation has been reported to play essential roles in muscle biology. It represses transcription by blocking the binding of transcription factors and promoting the formation of an inactive compact chromatin structure (Picard et al., 2014). DNMT1, one of DNMT family members, is differentially expressed in the muscle of pigs with different fat contents, whereas DNMT3b is a known factor affecting beef quality traits (Zhang et al., 2014). It was reported that *MYF6* is highly expressed and hypermethylated in lean pigs compared to high-fat pigs (Nogalski et al., 2018). An SNP in the *ELOVL6* gene is associated with an increase in methylation and a decrease of its expression, which may result in the different fatty acid contents in muscle and backfat (Li and Durbin, 2010). Moreover, methylation on pectoral muscle tissues has a highly significant effect on muscle fiber density and drip loss in broilers (Farke et al., 2008). Methylation affected the expression of *FGF2* in the leg muscles of broiler, which is related to myoblast proliferation and meat quality (Anderson et al., 2012). Hypermethylation in the downregulation of *HK-2* and *PFKFB4* decreased glycolytic potential in the psoas major (Goncalves et al., 2018). Additionally, hypomethylation in the upregulation of miR-378 silences the expression of the target genes and promotes capillary biosynthesis in the muscle (Goncalves et al., 2018). Importantly, DNA methylation plays a role during myogenic differentiation. During differentiation of the myoblast global DNA methylation levels increase, and hypermethylation is associated with genes involved in muscle contraction processes (Miyata et al., 2015), and coordinately regulate genes during myogenic differentiation (Miyata et al., 2015; Laker and Ryall, 2016). Collectively, DNA methylation definitely involves in meat quality, and it could be a major epigenetic regulator of beef quality.

MBD-seq, compared to other methods, is a cost-effective way to evaluate DNA methylation on the whole genome. Its strategy is to reduce the complexity of the genome by enrichment of methylated DNA with MethylCap protein against 5-methylcytosine. But it likely produces a bias toward the high-CpG-dense regions because the highly methylated regions are preferentially captured. That was why we performed step-wise elution for the capture of the methylated DNA to stratify the genome into different methylated CpG fractions (Brinkman et al., 2010). DNA fragments with only a few methyl-CpGs are found in the low salt elution, while fragments containing many methyl-CpGs are only eluted at the high-salt. Step-wise elution facilitates the detection of differentially methylated regions not only within the highly CpG dense regions like CpG islands but also in the regions with lower CpG density such as non-CpG islands and other regions. Using this approach, we acquired high sequence coverage during sequencing. We analyzed the data of the high, medium, and low methylated regions, respectively, to assess DNA methylation variations between the tender and tough groups. In the present study, the exons had a much higher degree of methylation than the other regions, and around 60% of the peaks are located in the intergenic regions. Additionally, we observed that the methylation of exons correlates highly and negatively with the gene expression in beef. All these results support our hypothesis that DNA methylation is involved in beef tenderness variation.

In annotation corresponding to DMRs, the ATP binding cassette family is the most important gene sets. This family, also known as the ABC superfamily, encodes proteins that use ATP as an energy resource to transport substrates across the cell membranes. Its members are essential for many processes in the cell and are thought to participate in the absorption and secretion of endogenous and exogenous substances and muscle regeneration (Bunting, 2002). More than 100 ABC transporters are identified from prokaryotes to humans, of which, more than 30 have been reported in cattle. For example, the expression of *ABCB1* and *ABCG2* were detected in the rumen (Anders and Huber, 2010). Researches in cattle found that ABC transporters mainly function on lipid transport. *ABCA1*, *ABCG1*, and *ABCA7* are differentially expressed between the lactation and non-lactating stages, showing species-specific patterns in mammary tissue (Mani et al., 2010). The enhanced expression for *ABCA1* and decreased expression for *ABCA7*, *ABCG2* was observed in bovine mammary tissue during the dry period of lactation (Farke et al., 2008). The presence of *ABCAS*, *ABCA1*, and *ABCG1* are involved in muscle activity and mammary epithelial cells (Dean et al., 2001; Mani et al., 2011). However, very few reports on ABC transporters were related to beef quality. In our study, A total of 21 members of ATP binding cassette subfamily were identified as being differentially methylated between the tender and tough beef, and they include: *ABCA3*, *ABCA4, ABCA5, ABCA6, ABCA10, ABCA12, ABCA13, ABCB7, ABCB9, ABCB10, ABCC1, ABCC3, ABCC5, ABCC6, ABCC9, ABCD1, ABCD3, ABCD4, ABCF3, ABCG1, ABCG2*. Differential methylation of these ABC transporters may influence the extent of lipid transport and result in fatty acid metabolism of the *longissimus lumborum*, consistent with our previous results that fat contents of the tender and tough beef differ (Zhao et al., 2012b).

Importantly, myosin related genes were another ones differentially methylated between these two kinds of beef. Myosins are a superfamily of motor proteins known for their roles in muscle contraction. The structure and function of myosins are globally conserved across species. Some isoforms of myosins have specialized functions in certain cell types, such as muscle. Some researchers have linked myosins with meat tenderness (Goncalves et al., 2018). Myosin light chain 1 was reported as a potential indicator of proteolysis and tenderness of beef when it is released from myofibrillar fraction during postmortem aging (Anderson et al., 2012). And myosin heavy chain IIX was found inversely related to beef tenderness (Picard et al., 2014). Additionally, *MyHC-I*, *MyHC-IIa*, and *MyHC-IIx* exhibit different expression patterns across various locations of skeletal muscle, and expression levels are negatively related to beef tenderness (Zhang et al., 2014). These, however, are contrary to our previous results that *MYH3* and *MYH8* are positively associated with beef tenderness (Zhao et al., 2012b). Therefore, the relationships between MyHC and tenderness are variable, implying that other environmental or epigenetic factors may play roles in the regulation of MyHC and beef tenderness (Maltin et al., 2003; Chriki et al., 2012). Herein we observed myosin-related genes were differentially methylated between the tender and tough beef. They belong to three categories, namely myosins (*MYO5A, MYO10, MYO15A, MYO16, MYO18B, MYO19, MYO1B, MYO1C, MYO1D, MYO1F, MYO1G, MYO3B, MYO5B, MYO7A, MYO7B, MYO9B*), myosin heavy chains (*MYH10, MYH14, MYH6, MYH7B, MYH8, MYH9*), myosin light chains (*MYL3, MYLK, MYLK4*). *MYH8* was only found differentially methylated while distinctly expressed in the tender and tough beef. The other myosins were not differentially expressed, suggesting that DNA methylation effects may act upon more distal genes, therefore may be too subtle to detect using our approach, or other mechanisms may be involved in.

Many factors influence beef quality and tenderness, such as breeds, production style, sex, slaughtering age, welfare before slaughter, and other environmental impacts, including temperature, humidity, management, nutrition, *etc*. Even in the same animal, meat quality varies among different parts of the carcass. Additionally, postmortem factors, affecting the conversion of muscle to meat, also contribute to the variation of beef tenderness (Maltin et al., 2003; Hocquette et al., 2012; Bonny et al., 2018). Our study was conducted using steers of Angus from one cut of *longissimus lumborum* only. Therefore, further research needs to be conducted to explore the biological effects of epigenetic factors regulating beef quality and tenderness.

## Conclusion

In this study, we report the DNA methylome profiling in divergent tenderness of beef. We found that methylations were mainly observed on the intron and exon of genes. Differential patterns of DMRs were identified between the tender and tough beef. Based on the selected DMRs, ATP binding cassette subfamily and myosin-related genes were highly methylated gene sets. Generation of neurons, GTPase activity, ion transport, and anion transport were the most profoundly affected pathways related to beef tenderness. Meanwhile, we also explored the relationship between DNA methylation and gene expression, implying that *MYH8, NAALAD2, PLA2G4A, UHRF1* were the most likely candidate biomarkers for beef tenderness. Overall, this first study of DNA methylome on beef tenderness may provide more in-depth insight into the mechanisms regulating meat quality, which will help us develop new strategies of beef genetics and breeding.

## Data Availability Statement

The datasets generated for this study can be found in The Angus cattle methylome data have been deposited into the NCBI Gene Expression Omnibus (http://www.ncbi.nlm.nih.gov/geo/query/acc.cgi?acc=GSE127215).

## Ethics Statement

The animal study was reviewed and approved by the University of Maryland Institutional Animal Care and Use Committee (Protocol # R-07-05). Written informed consent was obtained from the owners for the participation of their animals in this study.

## Author Contributions

JS conceived and designed the experiments. CZ, YL, JS, and FT performed the experiments. GJ and JC analyzed the data. CZ, GJ, JC, RB, LZ, and JS wrote the manuscript. All the authors contributed to the article and approved the submitted version.

## Conflict of Interest

GJ was employed by the company Shenzhen GenDo, Co., Ltd.

The remaining authors declare that the research was conducted in the absence of any commercial or financial relationships that could be construed as a potential conflict of interest.

## Abbreviations

HTO: high methylated-DNA from tough beef
LTE: low methylated-DNA from tender beef
LTO: low methylated-DNA from tough beef
MTE: medially methylated-DNA from tender beef
MTO: medially methylated-DNA from tough beef
HTE: high methylated-DNA from tender beef.
