# Supplementary material for: The Profiling of DNA Methylation and Its Regulation on Divergent Tenderness in Angus Beef Cattle
Source: Front Genet. 2020 Aug 26;11:939. doi: 10.3389/fgene.2020.00939 (PMC7479246; doi:10.3389/fgene.2020.00939)
Supplement: Supplementary file 1 [file Data_Sheet_1.PDF]

The Profiling of DNA Methylation and its Regulation on Divergent Tenderness in Angus Beef Cattle

Chunping Zhao[1, 2], Guanyu Ji[3], José A. Carrillo[2], Yaokun Li[1, 2], Fei Tian[2], Ransom L. Baldwin, VI[4], Linsen Zan[1] and Jiuzhou Song[2]*

[1]College of Animal Science and Technology, Northwest A&F University, Yangling, Shaanxi 712100, China; zhao.chunping@nwafu.edu.cn (CZ); liyaokun1986@163.com (YL); zanlinsenn@163.com (LZ)

[2]Department of Animal & Avian Sciences, University of Maryland, College Park, MD 20742, USA; carrillo@umd.edu (JC); tianfeifirst@gmail.com (FT); songj88@umd.edu (JS)

[3] Shenzhen Gene Do Health Sci&Tech Ltd., Shenzhen, 518083, China; jiguanyu@hotmail.com (GJ)

[4] Bovine Functional Genomic Laboratory, Animal and Natural Resources Institute, USDA-Agricultural Research Service, Beltsville, MD 20705-2350, USA; Ransom.Baldwin@ars.usda.gov (RB)

*Correspondence: songj88@umd.edu; Tel: 301-405-5943

Supplementary information

Table S1: Summary of reads of MBD-seq and alignments to the reference genome for the 12 elutes.

Sample	Raw Reads	Raw data(GB)	Mapped reads	Map rate (%)	Total mapped reads
**HTE_1**	9,985,933	0.50	9,031,429	90.44	35,257,025
**HTE_2**	10,373,992	0.52	9,575,629	92.30	
**HTO_1**	9,558,150	0.48	8,854,114	92.63	
**HTO_2**	8,451,362	0.42	7,795,853	92.24	
**MTE_1**	12,792,217	0.64	11,889,327	92.94	52,946,431
**MTE_2**	14,198,567	0.71	12,635,437	88.99	
**MTO_1**	15,927,645	0.80	14,981,895	94.06	
**MTO_2**	14,210,830	0.71	13,439,772	94.57	
**LTE_1**	9,403,974	0.47	8,162,432	86.80	46,738,093
**LTE_2**	13,201,186	0.66	12,051,816	91.29	
**LTO_1**	14,726,751	0.74	13,916,976	94.50	
**LTO_2**	13,441,211	0.67	12,606,869	93.79	
**Total**	146,271,818	7.31	134,941,549	92.25	134,941,549

HTE: **H**igh concentration salt eluted methylated-DNA from **te**nder beef

HTO: **H**igh concentration salt eluted methylated-DNA from **to**ugh beef

MTE: **M**edium concentration salt eluted methylated-DNA from **te**nder beef

MTO: **M**edium concentration salt eluted methylated-DNA from **to**ugh beef

LTE: **L**ow concentration salt eluted methylated-DNA from **te**nder beef

LTO: **L**ow concentration salt eluted methylated-DNA from **to**ugh beef

Table S2: Summary of mapped reads for the tender and tough beef

**Tender**	**Mapped reads**	**Tough**	**Mapped reads**
**HTE_1**	9,031,429	**HTO_1**	8,854,114
**HTE_2**	9,575,629	**HTO_2**	7,795,853
**MTE_1**	11,889,327	**MTO_1**	14,981,895
**MTE_2**	12,635,437	**MTO_2**	13,439,772
**LTE_1**	8,162,432	**LTO_1**	13,916,976
**LTE_2**	12,051,816	**LTO_2**	12,606,869
**Total**	63,346,070	**Total**	71,595,479

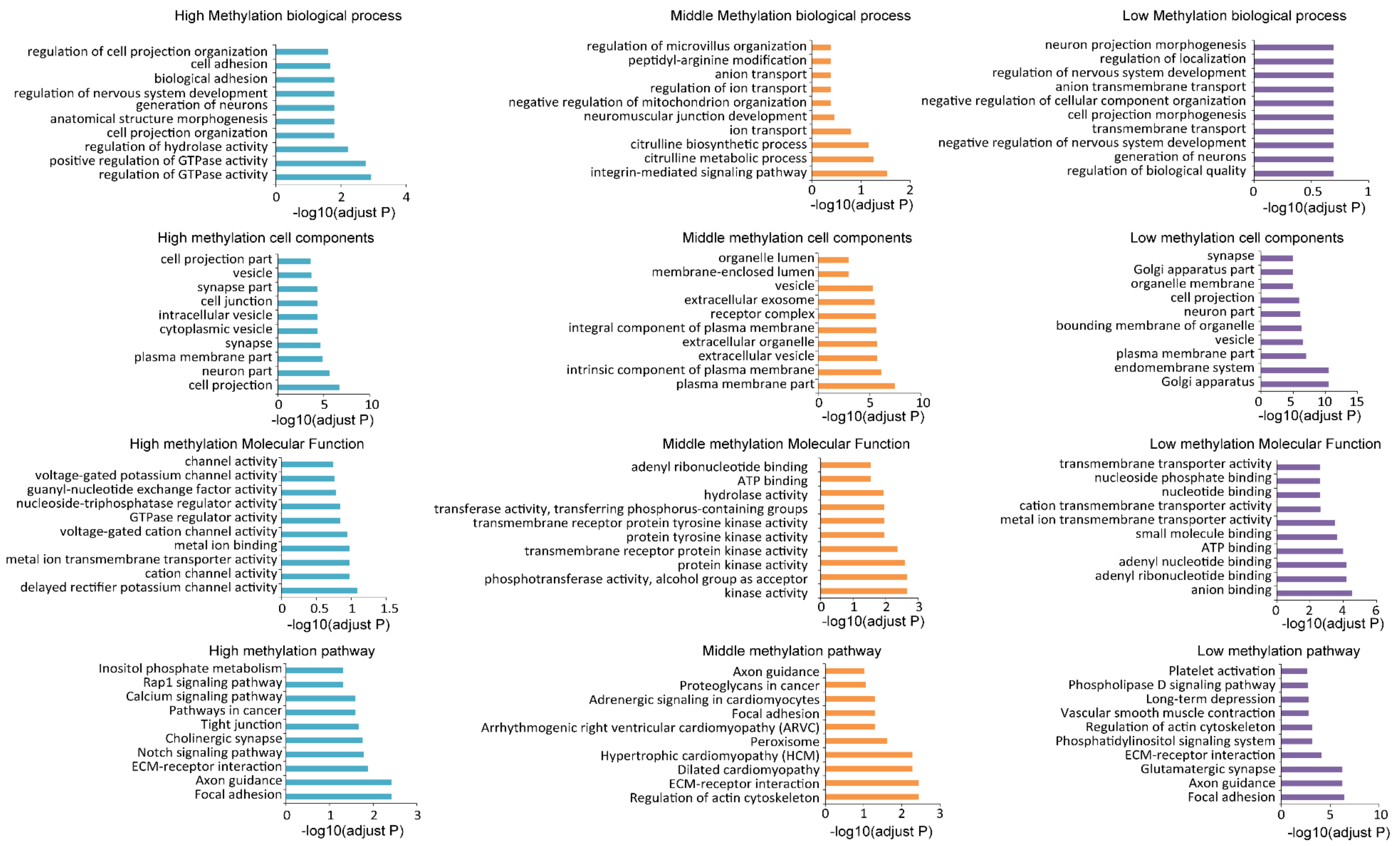

FigureS1: GO term analysis and KEGG analysis results. The first line showed the results from high methylation regions; the second line for medium methylation regions and the third line for low methylation regions. BP: biological process; CC: cellular component; MF: molecular function.

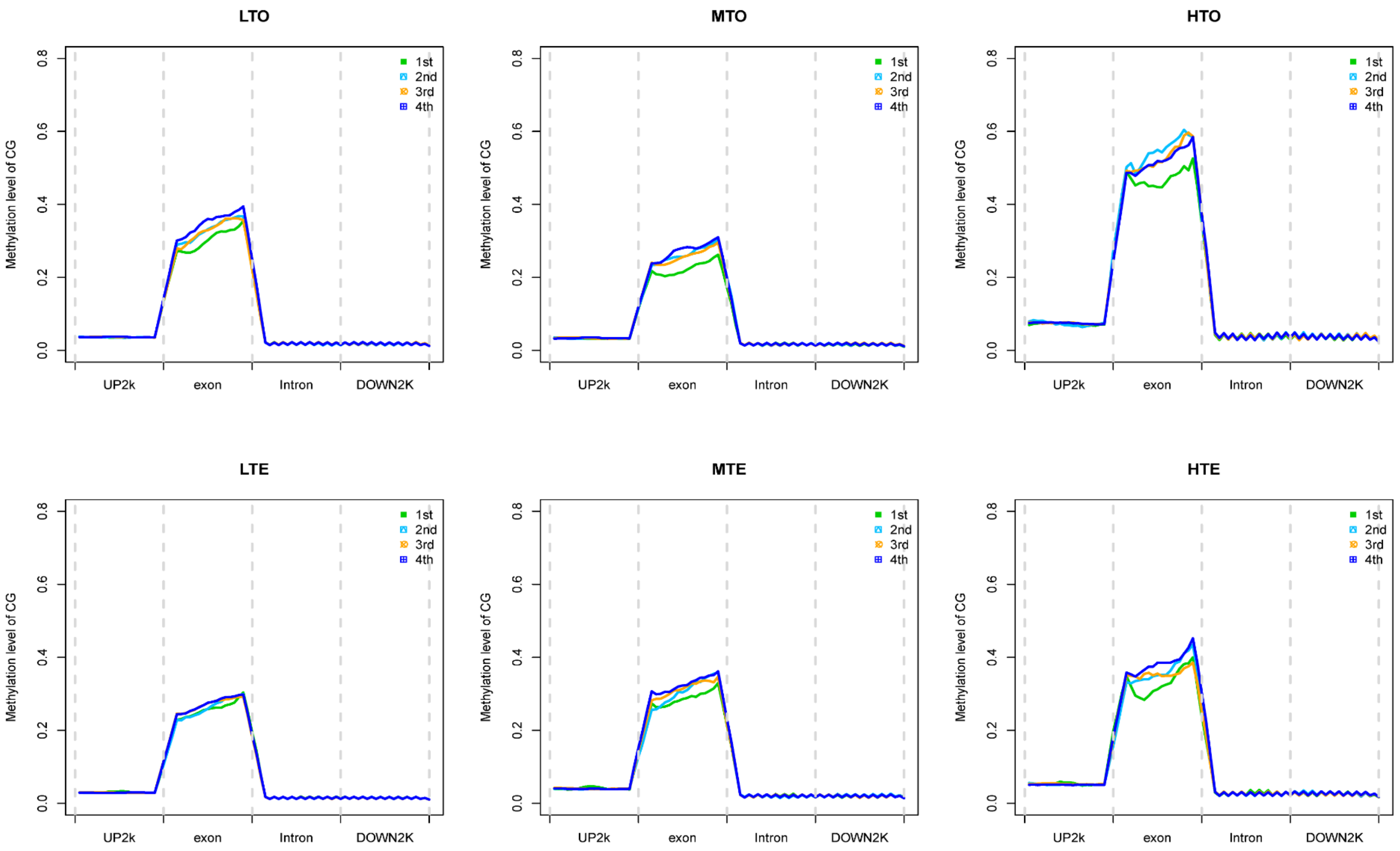

Figure S2: The methylation level of genes in four expression classes.
